# Hydrogenated Boron Phosphide THz-Metamaterial-Based Biosensor for Diagnosing COVID-19: A DFT Coupled FEM Study

**DOI:** 10.3390/nano12224024

**Published:** 2022-11-16

**Authors:** Chunjian Tan, Shaogang Wang, Huiru Yang, Qianming Huang, Shizhen Li, Xu Liu, Huaiyu Ye, Guoqi Zhang

**Affiliations:** 1 Electronic Components, Technology and Materials, Delft University of Technology, 2628 CD Delft, The Netherlands; 2 Engineering Research Center of Integrated Circuits for Next-Generation Communications, Ministry of Education, School of Microelectronics, Southern University of Science and Technology, Shenzhen 518055, China

**Keywords:** hydrogenated boron phosphide monolayer, terahertz metamaterial, bowtie triangle ring microstructure, COVID-19 diagnosis

## Abstract

Recent reports focus on the hydrogenation engineering of monolayer boron phosphide and simultaneously explore its promising applications in nanoelectronics. Coupling density functional theory and finite element method, we investigate the bowtie triangle ring microstructure composed of boron phosphide with hydrogenation based on structural and performance analysis. We determine the carrier mobility of hydrogenated boron phosphide, reveal the effect of structural and material parameters on resonance frequencies, and discuss the variation of the electric field at the two tips. The results suggest that the mobilities of electrons for hydrogenated BP monolayer in the armchair and zigzag directions are 0.51 and 94.4 cm^2^·V${}^{-1}$·s${}^{-1}$, whereas for holes, the values are 136.8 and 175.15 cm^2^·V${}^{-1}$·s${}^{-1}$. Meanwhile, the transmission spectra of the bowtie triangle ring microstructure can be controlled by adjusting the length of the bowtie triangle ring microstructure and carrier density of hydrogenated BP. With the increasing length, the transmission spectrum has a red-shift and the electric field at the tips of equilateral triangle rings is significantly weakened. Furthermore, the theoretical sensitivity of the BTR structure reaches 100 GHz/RIU, which is sufficient to determine healthy and COVID-19-infected individuals. Our findings may open up new avenues for promising applications in the rapid diagnosis of COVID-19.

## 1. Introduction

Since the end of 2019, a continuous breakout of coronavirus disease 2019 (COVID-19, named by the World Health Organization) associated with a novel pathogenic CoV (SARS-CoV-2, first recognized in Wuhan city, Hubei province of China) spread around the world, which has put various countries on the highest alert and destabilized the global economics. As of October 2022, more than 600 million COVID-19-confirmed cases and over 6.4 million associated deaths have been reported [1]. A published study has clearly shown that asymptomatic infections exhibit a high percentage in the tested individuals, which means a potential risk of transmission of asymptomatic infections in the community [2]. In addition, asymptomatic patients as a major source of COVID-19 transmission have been a mainstream point [3]. Therefore, there is an urgent need for rapid, accurate, large-scale, and cost-effective detection methods or techniques for COVID-19. In particular, it is essential to monitor the asymptomatic cases in real time to avoid or reduce cluster outbreaks and spread. Among current biological-based technologies, the polymerase chain reaction (PCR) approach, as the “gold-standard” for viral detection, has been widely used for identifying SARS-CoV-2. However, it would take more than four hours to obtain the testing results. Most importantly, there is a risk of eliciting false-negative and false-positive results for the real-time PCR method [4]. Furthermore, although a tremendous amount of detection approaches with varying efficiency for COVID-19 have emerged, these approaches are not always in accordance with the real-time PCR method [5,6,7,8]. Accordingly, seeking more safety and efficient COVID-19 detection methods is of utmost importance for the prevention and control of epidemics, especially in the diagnosis of asymptomatic cases.

Terahertz time-domain spectroscopy (THz-TDS) has become an emerging analysis technique in the biomedical field because of the immediate, label-free, and non-destructive properties in the characterization process [9], and in the analysis of viruses and pathogens in particular [10,11,12]. By integrating the THz technique with metamaterials, THz-metamaterial biosensors can be developed, which are highly sensitive to the subtle changes in the surrounding environment [9,13,14]. Furthermore, the resonance frequency or multi-resonance feature of metamaterials in the THz regime can be controlled by adjusting the structural parameters, material characteristics, and dielectric constant of the environment. Consequently, they are highly flexible and tunable in terms of optical properties [15,16]. At the same time, in the context of biosensing, the roadmap for THz metamaterial sensing moves towards high sensitivity, low limit of detection, high specificity, ease of preparation, and miniaturization. THz metamaterial technologies will be the key technologies in future sensing platforms.

Unfortunately, THz metamaterial sensing is still facing several challenges [17,18]: (i) The limited tunability of metals in optical constants, which hinders the sensitivity improvement and development of THz biosensors. (ii) For THz waves, the metallic geometries at a deep subwavelength scale are difficult to design through economical or industrial methods such as shade/etching techniques. (iii) The intrinsic loss of materials. Upon THz range, metals are treated as lossy dielectrics, which causes significant losses in the resonant loops. This will very likely increase the possibility of damaging the double negative band. As a result, exploring alternative material platforms for enhancing the sensing parameters of THz biosensors is imperative. The use of two-dimensional (2D) materials to solve these limitations is a new wave of revolutionary in THz metamaterial sensing. Owing to its fascinating tunability in electrical and optical properties, carbon-based materials, especially monolayer graphene, has been widely investigated in THz sensing [19]. Meanwhile, other 2D materials, such as black phosphorus (BP) and molybdenum disulfide (MoS^2^), have also been incorporated into THz metamaterial technology because of similar physical properties [20,21]. It inspires us that 2D materials can be a suitable platform for replacing conventional metals in the future. Furthermore, metamaterial research is gradually turning towards metasurfaces, namely the 2D counterpart of volumetric metamaterials. Accordingly, exploring more 2D materials as metamaterials or metasurfaces in THz sensing technologies could be an excellent option to meet the future need for label-free, high specificity, repeatable, and rapid detection techniques.

Hydrogenated boron phosphide monolayer, a graphane-like ternary compound, is a wide-bandgap (3.76 eV) semiconductor with a buckled honeycomb structure [22]. It has received great interest in energy storage, energy conversion, and nanoelectromechanical devices due to its outstanding stability, elastic properties, and lower thermal conductivity [23,24]. At present, none of the studies report the successful synthesis of hydrogenated boron phosphide monolayer at the experimental level. Nevertheless, some researchers have confirmed that its dynamic stability is stronger than that of graphene and silicane at the theoretical level [25,26]. Therefore, this provides the theoretical support for its applied research in optical biosensing, and THz metamaterial biosensing in particular. Notably, the THz metamaterial biosensor based on the hydrogenated boron phosphide monolayer is still lacking. Accordingly, the possibility of its THz metamaterial in the application of COVID-19 diagnosis is worth exploring to offer theoretical references for the fundamental research in THz metamaterials based on 2D materials with surface functionalization.

In this work, we construct hydrogenated boron phosphide-based bowtie triangle ring microstructure, and discuss its response to different structure designs, carrier density, and the dielectric constants of the selected analyte, mainly focusing on the resonance frequency, electric field, and sensitivity. Meanwhile, the mobility of hydrogenated boron phosphide is calculated by using the density functional theory method. The mobilities for electrons in the armchair and zigzag directions are 0.51 and 94.4 cm${}^{2}$·V${}^{-1}$·s${}^{-1}$, respectively, while for holes are 136.8 and 175.15 cm^2^·V${}^{-1}$·s${}^{-1}$, respectively. Furthermore, it is found that the resonance frequency of the microstructure is highly sensitive to the variation of structure, carrier density, and permittivity of the analyte. The transmission spectra exhibit a red-shifted behavior with the increase in the overall length of the microstructure, and the electric field at the two tips obviously weakens in the meantime. Eventually, the theoretical sensitivity of the microstructure turned out to be 100 GHz/RIU.

## 2. Theoretical Model and Computational Methodologies

All density functional theory (DFT) calculations are performed within the framework of the Vienna Ab-initio Simulation Package (VASP) [27]. The hydrogenated boron phosphide (BP) monolayer is modeled using an orthorhombic primitive unit cell to better impose strain along the zigzag and armchair directions. The ion-electron interaction is treated by adopting the projected-augmented wave (PAW) method [28]. Meanwhile, the Perdew–Burke–Ernzerhof (PBE) generalized gradient approximation is also used in the DFT calculations [29]. The kinetic energy for the wave function is set to 650 eV. The convergence criterion for geometric relaxation is <10${}^{-5}$ eV for the total energy difference and the remainder force on atom <0.005 eV/Å. In the 2D Brillouin zone, 9 × 11 × 1 and 15 × 21 × 1 k-point grids are applied for geometric optimization and static calculation, respectively. The slab monolayer is separated by a vacuum thickness of 15 Å to minimize artificial interactions between two adjacent images [30].

**Figure 1:**
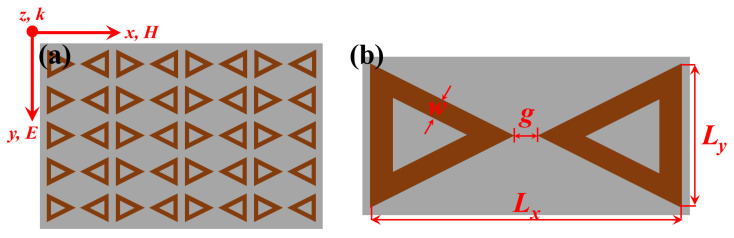
Schematic diagram of hydrogenated BP monolayer-based metasurface on the silicon substrate: (**a**) Periodic structure where the incident THz waves with *x*-polarization are along the *z*-direction; (**b**) The unit cell with geometrical parameters where $L_{x}$ varies with $L_{y}$, *g* = 2 μm, and *w* = 6 μm. The periodicity is set to $L_{x}$ + *w* and $L_{y}$ + *w* for *x* and *y* directions.

The schematic structure of the hydrogenated BP monolayer metasurface is illustrated in Figure 1. The unit cell comprises BTR of hydrogenated BP monolayer positioned on the top of a 500-μm-thick silicon substrate with the dielectric constant of ε = 11.9 and the constant conductivity σ = 0.004 S/m [9,31]. The unit cells based on hydrogenated BP monolayer are arranged in a periodic array in the x−y plane, as depicted in Figure 1a. *P* represents the periodicity of array patterns. The gap size between two tip-to-tip equilateral triangle rings and the corresponding ring width are designated as *g* and *w*, respectively. Notably, two tip-to-tip equilateral triangle rings are mirrored on the silicon substrate. The geometrical parameters of the investigated unit cell are described specifically in Figure 1b.

In our work, the thickness of the hydrogenated BP monolayer is set as 1.0 nm. The incident plane wave with *x*-polarization propagates vertically in a direction paralleling the *z*-axis to the hydrogenated BP monolayer metasurface. The transmission spectrum as a function of incident wavelengths and the electric field distributions at resonance peaks are calculated in the full-wave electromagnetic simulator COMSOL Multiphysics. In order to obtain the response characteristic of hydrogenated BP monolayer metasurface in the THz domain, we utilize an effective surface conductivity approach to characterize hydrogenated BP monolayer. Theoretically, the surface conductivity of hydrogenated BP monolayer can be approximated as Drude model [32,33,34]

(1) $$\sigma \left(w\right)=i\frac{ne^{2}}{m^{*}\left(w+i\Gamma \right)}$$

where *w*, *e*, *n*, and $m^{*}$ are the angular frequency of the incident wave, electron charge (1.6 $\times \;10^{-19}$ C), carrier concentration, and effective carrier mass, respectively. Unless otherwise stated, *n* = 1 ×$10^{17}$ cm${}^{-2}$ is used for all calculations. Γ is the damping constant, being the inverse of the intrinsic relaxation time. The intrinsic relaxation time is calculated by $\tau =\left(\mu \mu_{c}\right)/\left(ev_{F}^{2}\right)$ where μ and $v_{F}$ are carrier mobility and Fermi velocity. The chemical potential is determined by $\mu_{c}=\hbar v_{F}\sqrt{\pi n}$. Moreover, the Fermi velocity is deduced through $v_{F}=\sqrt{E_{F}/\left(2m^{*}\right)}$ in which $E_{F}$ is the Fermi energy and the corresponding value is 3.88 eV for the hydrogenated BP monolayer. To summarize, the equivalent permittivity of the hydrogenated BP monolayer can be deduced by the surface conductivity as [33]

(2) $$\varepsilon_{2D}=1+\frac{i\sigma (w)}{w\varepsilon_{0}t_{m}}$$

where $\varepsilon_{0}$ is the vacuum permittivity and $t_{m}$ is the layer thickness of 2D materials.

## 3. Results and Discussion

Before beginning to theoretically decipher the sensing performance of the hydrogenated BP monolayer-based THz sensing device, the carrier mobility of the hydrogenated BP monolayer should be calculated to obtain the corresponding dielectric constant in the desired THz region. Carrier mobility usually refers to the overall movement speed of electrons and holes in the semiconductors, being an important physical quantity to judge the performance of semiconductor devices. In 2004, the successful exfoliation of graphene set off a wave of research into 2D materials and their physicochemical properties. The high carrier mobility exhibited by 2D materials such as graphene and black phosphorus is one of the key research and application hotspots, and a tremendous amount of related studies have been performed on theoretical calculations. As electrons are not only subjected to external E-field forces during their movement, but also constantly collide with the lattice, impurities, and defects in an irregular manner. This greatly increases the difficulty of the theoretical calculations. At present, the commonly applied theories for calculating the carrier mobility of 2D materials are the deformation potential and Boltzmann transport theories. For the deformation potential theory, some factors such as lattice vibration and electron-electron interaction are not taken into account, resulting in the existence of calculation errors. The Boltzmann transport theory carefully treats the electron-electron interaction and utilizing the first-principles calculation and maximally localized Wannier functions interpolation method can deal with the calculation of carrier mobility. However, the disadvantage is that it is too computationally intensive and requires high computational costs.

In the present work, according to the deformation potential theory, the carrier mobility $\mu_{2D}$ of hydrogenated BP monolayer, with a view of revealing the migration characteristics of electrons and holes, can be obtained by the formula [35]

(3) $$\mu_{2D}=\frac{e\hbar^{3}C_{2D}}{k_{B}Tm^{*}m_{d}E_{1}^{2}}$$

where $m^{*}$ is the effective carrier mass in the transport direction, T is the temperature that is set to 300 K in this study, $k_{B}$ is the Boltzmann constant (≈1.38 $\times 10^{-23}$ J/K). $E_{1}$ represents the deformation potential constant for electrons clustered at the CBM or for holes at the VBM along the transport direction, being determined by $E_{1}=△E/\left(△l/l_{0}\right)$, in which △E indicates the energy changes of CBM and VBM under compressive or tensile strain, $l_{0}$ is the lattice constant of 2D materials in the transport direction, △l is the amount of deformation of lattice constant by uniaxial strain. $m_{d}$ is the average effective mass of carriers, which is defined as $m_{d}=\sqrt{m_{*}^{x}m_{*}^{y}}$. In the 2D materials, the elasticity modulus can be expressed as $C_{2D}=2\left[\partial^{2}E/\partial {\left(△l/l_{0}\right)}^{2}\right]/S_{0}$ where *E* is the total energy and $S_{0}$ is the area of the xy-plane after relaxation.

The values of $C_{2D}$ of hydrogenated BP monolayer along the zigzag (*x*) and armchair (*y*) directions are 103.96 J/m${}^{2}$ and 103.85 J/m${}^{2}$, respectively. The $E_{1}$ for electrons (holes) of hydrogenated BP monolayer along the x and y directions are 8.42 eV and 9.14 eV (5.92 eV and 5.71 eV), respectively. This means that the $E_{1}$ is independent of the transport direction but of the type of carrier. The $m^{*}$ of electrons (holes) for hydrogenated BP monolayer in the x and y directions are 0.163 m${}_{e}$ and 25.355 m${}_{e}$ (0.555 m${}_{e}$ and 0.763 m${}_{e}$), respectively, which indicates that there is an anisotropic transport feature for electrons and holes. However, the divergence in the anisotropy of electrons is more tremendous than in holes. By substituting the calculated $C_{2D}$ and $E_{1}$ into Equation (3), the carrier mobility of the hydrogenated BP monolayer is obtained. All of those calculated results are listed in Table 1. For comparison, the corresponding parameter values of the BP monolayer without hydrogen passivation are also shown. It can be found that the hydrogenation of the BP monolayer has an obvious influence on its carrier mobility and enhances the anisotropic divergence in carrier transport.

**Figure 2:**
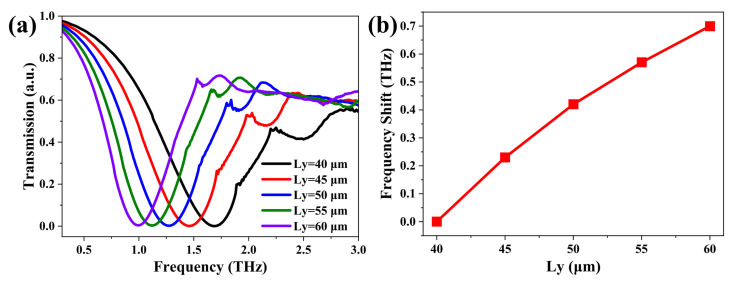
(**a**) Normalized THz transmission amplitudes of the investigated BTR with different $L_{y}$. (**b**) The resonance-frequency shift as a function of $L_{y}$.

In order to explore the design window of BTR with hydrogenated BP monolayer, we investigate the influence on it by modulating the structural parameter ($L_{y}$) shown in Figure 1b. In this simulation, $L_{y}$ is regulated in an increment of 5 μm from 40 μm to 60 μm, while *w* and *g* are set to 6 μm and 2 μm, respectively. In Figure 2a, the simulated transmission spectra clearly present that there is only one resonance peak for the BTR with hydrogenated BP monolayer. Meanwhile, the resonant frequency of BTR moves toward the lower frequency region (i.e., red-shift) with the increasing $L_{y}$. The underlying cause may be the increasing of the equivalent inductance with the length of BTR with a hydrogenated BP monolayer. Moreover, the corresponding resonant frequencies are 1.69 THz, 1.46 THz, 1.27 THz, 1.12 THz, and 0.99 THz for 40 $L_{y}$m, 45 μm, 50 μm, 55 μm, and 60 μm, respectively. As plotted in Figure 2b, with the increase in $L_{y}$, there is a nearly linear increase in the resonance-frequency shift. However, it is noted that the resonance-frequency shift between two $L_{y}$ values gradually decreases, which indicates that adjusting the transmission properties through a larger $L_{y}$ value is not recommended for the BTR structure. The electric field distribution in the BTR structure further provides powerful evidence for this result. In Figure S1a–e, it can be found that the spot size of the electric field in the tips of two equilateral triangle rings becomes smaller as the length $L_{y}$ increases. This means that the enhancement of the electric field clustered at the tips is decreasing. Apart from an increasing electric field for $L_{y}$ = 45 $L_{y}$m, the strength of the electric field at the tips decreases when the length $L_{y}$ is varied in a step size of 5 μm from 40 μm to 60 μm, as displayed in Figure S1f–j. This is also another underlying reason why a larger length $L_{y}$ value is not expected in the BTR structure.

**Figure 3:**
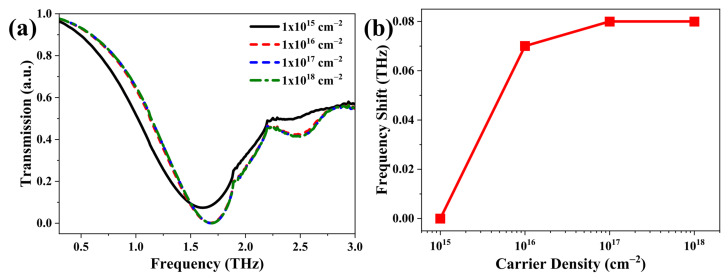
(**a**) Transmission spectrum with different carrier densities. (**b**) The resonance-frequency shifts versus carrier densities at the resonant frequencies of the BTR structure.

One of the most interesting features of 2D materials is that their conductivity depends largely on the chemical potential, which can be controlled by carrier density, as described in the section on the theoretical model and computational methodologies. In addition to the structural parameters of BTR, the carrier density of the hydrogenated BP monolayer is another critical factor that affects the performance of the BTR structure. Therefore, it is essential to explore the influence of carrier density (i.e., chemical potential) on transmission spectra of BTR structure with hydrogenated BP monolayer. In this simulation, four carrier densities, namely 1 ×$10^{15}$ cm${}^{-2}$, 1 ×$10^{16}$ cm${}^{-2}$, 1 ×$10^{17}$ cm${}^{-2}$, and 1 ×$10^{18}$ cm${}^{-2}$, are considered. The carrier densities with less than 1 ×$10^{15}$ cm${}^{-2}$ are also carefully tested, and there are no expected transmission spectra as is shown in Figure 3a (the corresponding results are not shown here). Figure 3 clearly clarifies the response of transmission spectra to the investigated carrier densities. We found that when the carrier density is changed from 1 ×$10^{15}$ cm${}^{-2}$ to 1 ×$10^{16}$ cm${}^{-2}$, the transmission spectra exhibit an obvious divergence and the resonant valley moves into the higher frequency regime. The resonant frequencies are 1.61 THz and 1.68 THz for 1 ×$10^{15}$ cm${}^{-2}$ and 1 ×$10^{16}$ cm${}^{-2}$, respectively. Unfortunately, the transmission spectra for the carrier density within the range of 1 ×$10^{16}$ cm${}^{-2}$ to ×10${}^{18}$ cm${}^{-2}$ are hardly indistinguishable. However, there exists a 0.01 THz difference between the values of 1 ×$10^{16}$ cm${}^{-2}$ and 1 ×$10^{17}$ cm${}^{-2}$. For the values of 1 ×$10^{17}$ cm${}^{-2}$ and 1 ×$10^{18}$ cm${}^{-2}$, the difference for their resonant frequencies is zero. It suggests that the upper limit is 1 ×$10^{17}$ cm${}^{-2}$ for adjusting the transmission properties of BTR through carrier density of hydrogenated BP monolayer. This result is further demonstrated by the resonance-frequency shifts, as plotted in Figure 3b. The resonance-frequency shift linearly increases with a relatively large slope rate within the range from 1 ×$10^{15}$ cm${}^{-2}$ to 1 ×$10^{16}$ cm${}^{-2}$. Meanwhile, there is a big resonance-frequency shift, which is expected in the THz sensing application. As a result, this range of carrier densities is exciting for the BTR structure. As shown in Figure S2, we reveal the response of the electric field to different density values. When the carrier density increases to 1 ×$10^{16}$ cm${}^{-2}$ from 1 ×$10^{15}$ cm${}^{-2}$, there is an obvious enhancement of electric field at the tip region. With further increases in carrier density, the electric field at two tips is not almost enhanced. The enhancement of the electric field for different densities is consistent with the result in resonance-frequency shifts.

Sensing performance analysis for COVID-19. Biologically, plasma and cells are the major portions of human blood. Plasma is a mixture including water, proteins (e.g., enzymes and albumin), and other dissolved substances, etc. For blood cells, the main ones are white blood cells, red blood cells, and platelets. In particular, white blood cells, containing lymphocytes, basophils, monocytes, neutrophils, and eosinophils are associated with defense against pathogens in the human body [37]. Some studies have demonstrated that the biochemistry and cellular composition of blood would be altered in COVID-19 patients compared to healthy individuals [38]. In the serum of patients with COVID-19 PCR-positive, some biochemical components (e.g., C-reactive proteins and alanine aminotransferase) would be obviously increased, whereas albumin is reduced [39,40]. Unlike viral infections, which usually result in high white blood cells and lymphocytes, the number and percentage of white blood cells and lymphocytes are remarkably lower in the COVID-19 confirmed cases [41,42,43]. Furthermore, the alteration in the counts of biochemical components such as lymphocytes and IgM antibodies would significantly change the dielectric constant of blood [44,45]. Therefore, it is hypothesized that the rapid diagnosis of COVID-19 could be achieved by detecting alterations in the permittivity of blood.

To investigate the sensing performance of BTR with hydrogenated BP monolayer, the blood of healthy individuals and COVID-19 patients are chosen as the target analytes. In actual experiments, the analyte is not evenly covered in the sensing region. However, in this simulation, blood samples are supposed to be a uniform blood plate with a thickness of 5 μm. As mentioned above, the alteration of blood components would result in a change in the permittivity of blood. The research has reported that the percentage of lymphocytes is decreased to less than 5% in most COVID-19 confirmed cases [46]. In addition, the dielectric constant is further reduced when the immune system is just beginning to produce immunoglobulin/antibody protein in response to COVID-19 antigen [47]. Therefore, the values of 1%, 3%, 5%, 7%, 9%, and 11% smaller dielectric constant compared to healthy blood are considered in this case. Dielectric properties can be characterized by the complex permittivity of materials. As a polar liquid, the complex permittivity of human blood can be approximately described by using the double Debye model within the THz frequencies. The complex permittivity includes two parts, namely real and imaginary parts, which are expressed as follows [48]

(4) $$\varepsilon^{{}^{\prime }}=\varepsilon_{\infty }+\frac{\varepsilon_{1}-\varepsilon_{2}}{1+{\left(w\tau_{1}\right)}^{2}}+\frac{\varepsilon_{2}-\varepsilon_{\infty }}{1+{\left(w\tau_{2}\right)}^{2}}$$

(5) $$\varepsilon^{{}^{”}}=\frac{\left(\varepsilon_{1}-\varepsilon_{2}\right)\left(w\tau_{1}\right)}{1+{\left(w\tau_{1}\right)}^{2}}+\frac{\left(\varepsilon_{2}-\varepsilon_{\infty }\right)\left(w\tau_{2}\right)}{1+{\left(w\tau_{2}\right)}^{2}}$$

where $\varepsilon_{\infty }$, $\varepsilon_{1}$, and $\varepsilon_{2}$ are the permittivity at the high-frequency limit, static limit permittivity, and intermediate dielectric value, respectively. *w* represents the angular frequency, which is deduced by 2πf (*f* is the frequency of incident wave). Moreover, $\tau_{1}$ and $\tau_{2}$ denote relaxation times of the first and second relaxation processes, respectively. These Debye parameter values for blood components within the range of 0.2 to 2.0 THz are listed in Table 2 [49]. The monopolar Debye parameters are extracted according to the amount of glucose concentration in blood plasma from 0 to 16,000 mg/dL [50].

**Figure 4:**
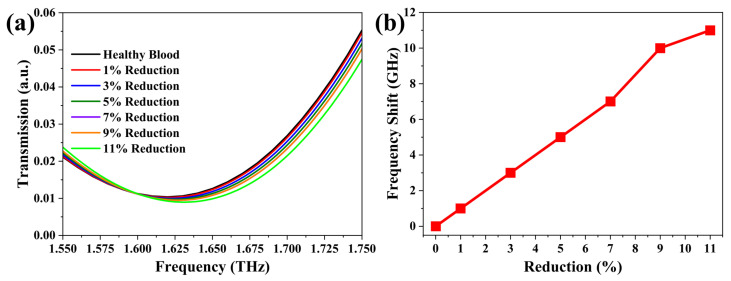
(**a**) Transmission spectra of BTR structure with hydrogenated BP monolayer and (**b**) the corresponding resonance-frequency shift at different permittivity reduction.

As illustrated in Figure 4a, the transmission spectra exhibit a slight blue-shift when the dielectric constant of whole blood is decreased in a step size of 2% within the range of 1% to 11%. The transmission valley has a minor reduction. With the reduction in permittivity of whole blood (see Figure 4b), i.e., gradually changing blood from normal status to unhealthy status, the resonance-frequency shift increases in a near linear relationship. The alteration of resonance frequency between permittivity reductions is within 3 GHz, which means that the BTR structure with a hydrogenated BP layer is difficult to respond to a smaller permittivity change and brings an obvious resonance-frequency shift. Furthermore, to reveal the effect of permittivity reduction on the electric field of the BTR structure, we also analyze the electric-field distribution, especially at the tip-to-tip region of two equilateral triangle rings. In comparison, the spots of the electric field at two tips of equilateral triangle rings without whole blood are significantly larger than that with whole blood, as displayed in Figures S1a and S3. For the cases with whole blood, the spot of the electric field for each value of permittivity reduction is almost unchanged, which is clearly plotted in Figure S3a–g. Meanwhile, the strength of the electric field (see Figure S3h–n) at the two tips is nearly the same, thus this further gives powerful evidence to the above discussion.

As an important element of the biosensors, sensitivity (*S*) can effectively characterize the sensing quality, being defined as

(6) $$S=\frac{△f}{△n}\left(\mathrm{GHz}/\mathrm{RIU}\right)$$

where △f is the difference between the reference frequencies with whole blood and the resonance frequency with a certain permittivity reduction. △n is the reduction percentage of the dielectric constant of whole blood. Note that these sensing parameters are calculated at the *w* = 6 μm and *g* = 2 μm. According to Equation (6), the sensitivity of the BTR structure with a hydrogenated BP monolayer for all reduction values turned out to be about 100 GHz/RIU. In order to better evaluate the sensing level of hydrogenated BP monolayer-based BTR structure, its sensitivity is compared with other previous studies. In comparison, we find that its sensitivity is close to 112.47 GHz/RIU in THz dielectric sensor for liquid crystal [51] and larger than 76.5 GHz/RIU in THz metamaterial biosensor for the detection of carcinoembryonic antigen [52]. However, compared to the sensitivity (498 GHz/RIU) of the BTR structure with metal materials, its sensitivity still needs improvement by redesigning the microstructure or changing the material parameters of hydrogenated BP monolayer, as well as other surface modification engineering [9]. In fact, the BTR structure could be equated to an inductance-capacitance oscillator. The changes in inductance or capacitance would induce different transmission spectra. In particular, the capacitance is related to the thickness of the BTR structure. Therefore, the 2D structure of the hydrogenated BP monolayer is the shortcoming in this structure, which leads to a very small contact area with the analyte. Some published studies have shown that bowtie-shaped tip-to-tip metallic prism MMs could generate strong electromagnetic interaction, which would lead to significant changes in the transmission spectra even though at a very low concentration [53,54,55]. Furthermore, because of its semiconductor characteristic and outstanding tunability in electronic properties, the hydrogenated BP monolayer-based BTR microstructure can adjust the transmission properties by only material parameters. Therefore, these features enable it to become a potential candidate in biosensing.

## 4. Conclusions

In summary, we theoretically describe the carrier mobility of hydrogenated BP monolayer and numerically analyze hydrogenated BP monolayer-based BTR microstructure by means of the DFT coupled FEM method. The DFT results show that the mobilities of electrons for hydrogenated BP monolayer in the armchair and zigzag directions are 0.51 and 94.4 cm${}^{2}$·V${}^{-1}$·s${}^{-1}$, whereas for holes the values are 136.8 and 175.15 cm${}^{2}$·V${}^{-1}$·s${}^{-1}$. Moreover, the response spectra can be controlled by changing the length of the BTR structure and carrier density of the hydrogenated BP monolayer. The theoretical sensitivity of BTR structure for COVID-19 reaches 100 GHz/RIU. Our work may offer new possibilities for exploiting 2D materials as sensing materials for COVID-19 diagnosis in the THz region. Moreover, other microstructures should be further designed and verified to inspire the performance of hydrogenated BP layers and high-accuracy detection.

## Figures and Tables

**Table 1:** Calculated carrier mobility ($\mu_{2D}$, cm${}^{2}$·V${}^{-1}$·s${}^{-1}$, deformation potential constant ($E_{1}$, eV), in-plane stretching modulus ($C_{2D}$,cJ/m${}^{2}$), and effective mass ($m^{*}$, $m_{e}$ is the electron mass) in BP monolayer without and with hydrogen passivation along zigzag and armchair directions at 300 K.

Treatment	Carrier Type	Direction	$E_{1}$	$C_{2D}$	$m^{*}$	$\mu_{2D}$
Without hydrogenation [36]	electron	zigzag	2.288	147.3746	0.198	1.017 × 10^4^
		armchair	2.223	147.3595	0.192	1.361 × 10^4^
	hole	zigzag	3.762	147.3746	0.180	4.493 × 10^3^
		armchair	3.856	147.3595	0.186	5.045 × 10^3^
With hydrogenation	electron	zigzag	8.42	103.96	0.163	94.4
		armchair	9.14	103.85	25.355	0.51
	hole	zigzag	5.92	103.96	0.555	175.15
		armchair	5.71	103.85	0.763	136.80

**Table 2:** Debye parameters of human blood in the 0.2–2.0 THz region.

Component	$\varepsilon_{\infty }$	$\varepsilon_{1}$	$\varepsilon_{2}$	$\tau_{1}$ (ps)	$\tau_{1}$ (ps)
Whole blood	2.1	130	3.8	14.4	0.1
Thrombus	2.2	130	3.7	16.1	0.1
Blood cells	3.4	2.5	23.8	410.8	1.8
Blood plasma	1.7	78.8	3.6	8.0	0.1
Water	3.3	78.8	4.5	8.4	0.1

## Data Availability

The data is available on reasonable request from the corresponding author.

## Supplementary Materials

The following supporting information can be downloaded at: https://www.mdpi.com/article/10.3390/nano12224024/s1, Figure S1: Distribution of electric field at the transmission valleys for different $L_{y}$. These valleys are corresponded to the frequencies: (a) and (f) 1.69 THz for 40 μm, (b) and (g) 1.46 THz for 45 μm, (c) and (h) 1.27 THz for 50 μm, (d) and (i) 1.12 THz for 55 μm, (e) and (j) 0.99 THz for 60 μm, respectively. Note that the rainbow range is set to 0 to 8 × 10${}^{6}$ to better observe the divergence of electric field between different $L_{y}$; Figure S2: Distribution of electric field at the resonant frequencies for different carrier densities. (a) and (e) 1.61 THz for 1 × 10^15^ cm${}^{-2}$, (b) and (f) 1.68 THz for 1 × 10^16^ cm${}^{-2}$, (c) and (g) 1.69 THz for 1 × 10^17^ cm${}^{-2}$, (d) and (h) 1.69 THz for 1 × 10^18^ cm${}^{-2}$. The rainbow range is from 0 to 8 × 10^6^; Figure S3: Distribution of electric field for the transmission valleys at different permittivity reduction. The rainbow is cut from 0 to 8 × 10^6^. Notably, (a) and (h) normal blood, (b) and (i) 1% reduction, (c) and (j) 3% reduction, (d) and (k) 5% reduction, (e) and (l) 7% reduction, (f) and (m) 9% reduction, (g) and (n) 11% reduction.
