# Supplementary material for: Hydrogenated Boron Phosphide THz-Metamaterial-Based Biosensor for Diagnosing COVID-19: A DFT Coupled FEM Study
Source: Nanomaterials (Basel). 2022 Nov 16;12(22):4024. doi: 10.3390/nano12224024 (PMC9697324; doi:10.3390/nano12224024)
Supplement: Supplementary file 1 [file nanomaterials-12-04024-s001.zip › nanomaterials-2028156-supplementary.pdf]

Supporting Information

Hydrogenated boron phosphide THz-metamaterial based biosensor for diagnosing COVID-19: A DFT coupled FEM study

Chunjian Tan[1,2], Shaogang Wang[1,2], Huiru Yang[2], Qianming Huang[2], Shizhen Li[2], Xu Liu[1,2], Huaiyu Ye[2,*], Guoqi Zhang[1,*]

[1] Electronic Components, Technology and Materials, Delft University of Technology, 2628 CD Delft, The Netherlands

[2] Engineering Research Center of Integrated Circuits for Next-Generation Communications, Ministry of Education, School of Microelectronics, Southern University of Science and Technology, Shenzhen 518055, China

* Correspondence: yehy@sustech.edu.cn and G.Q. Zhang@tudelft.nl

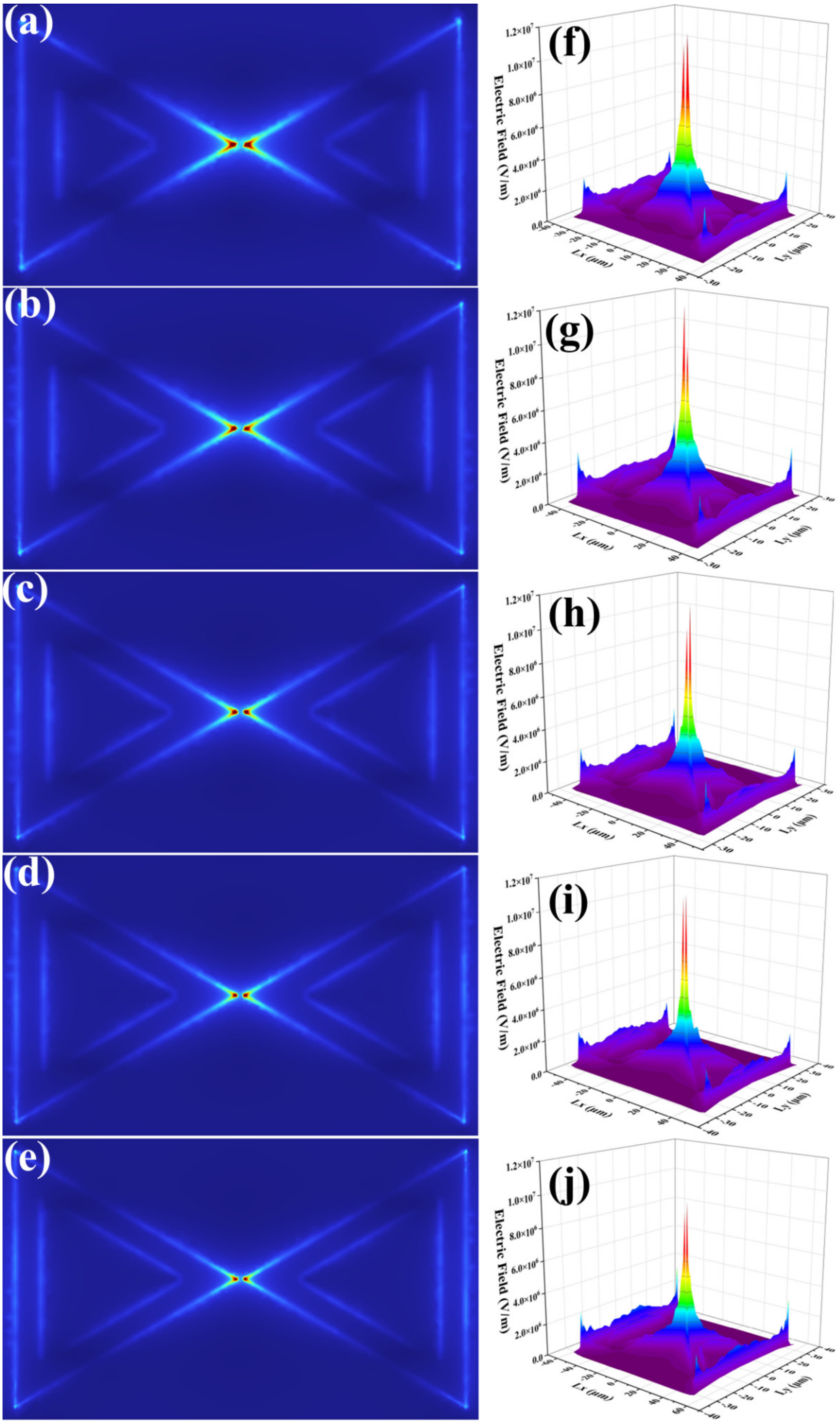

Figure S1. Distribution of electric field at the transmission valleys for different L_y. These valleys are

corresponded to the frequencies: (a) and (f) 1.69 THz for 40 μm, (b) and (g) 1.46 THz for 45 μm, (c) and (h) 1.27 THz for 50 μm, (d) and (i) 1.12 THz for 55 μm, (e) and (j) 0.99 THz for 60 μm, respectively. Note that the rainbow range is set to 0 to 8×10^6 to better observe the divergence of electric field between different L_y.

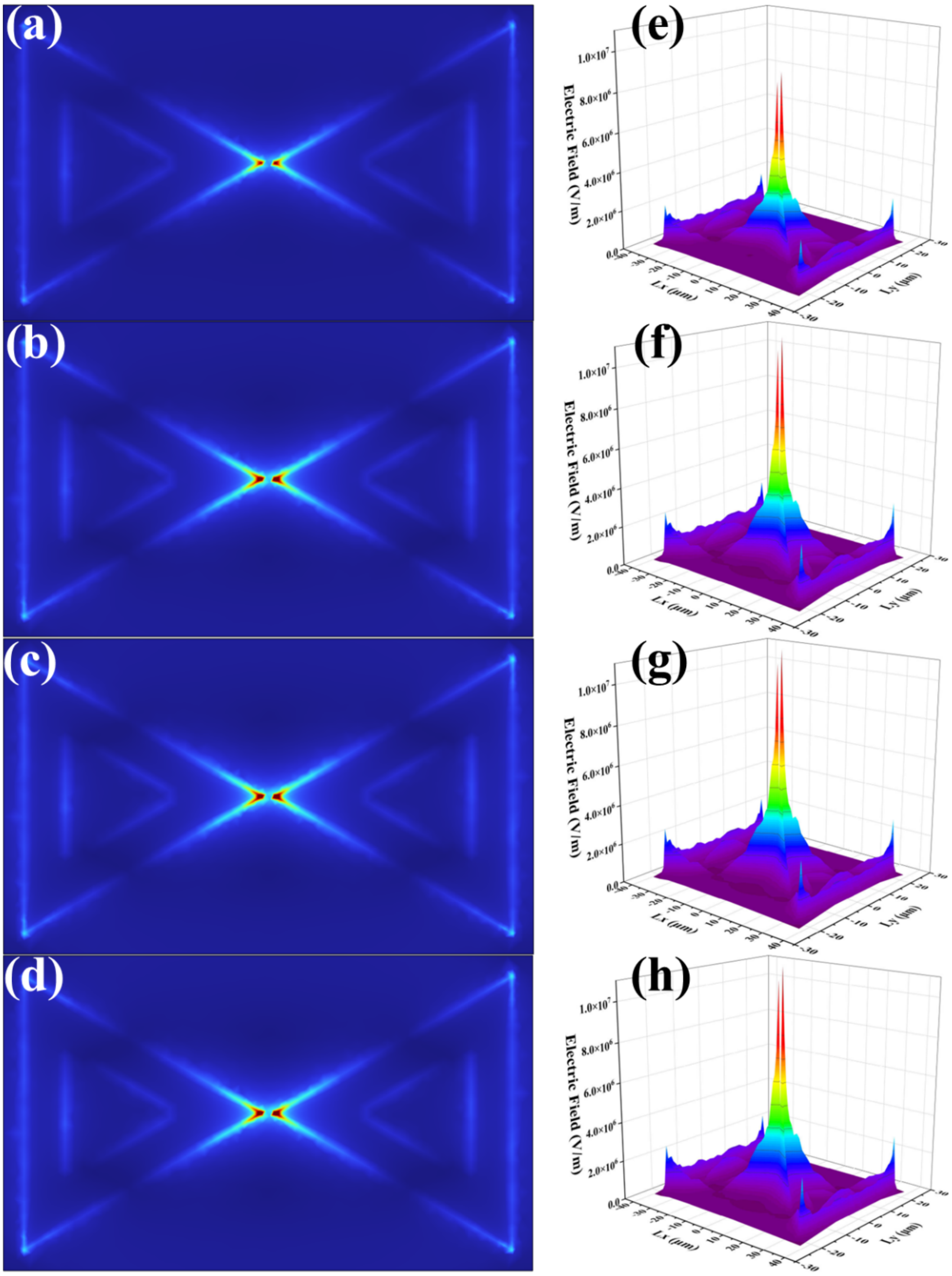

Figure S2. Distribution of electric field at the resonant frequencies for different carrier densities. (a) and (e) 1.61 THz for $1 \times 10^{15}\ cm^{-2}$, (b) and (f) 1.68 THz for $1 \times 10^{16}\ cm^{-2}$, (c) and (g) 1.69 THz for $1 \times 10^{17}\ cm^{-2}$, (d) and (h) 1.69THz for $1 \times 10^{18}\ cm^{-2}$. The rainbow range is from 0 to 8×10^6.

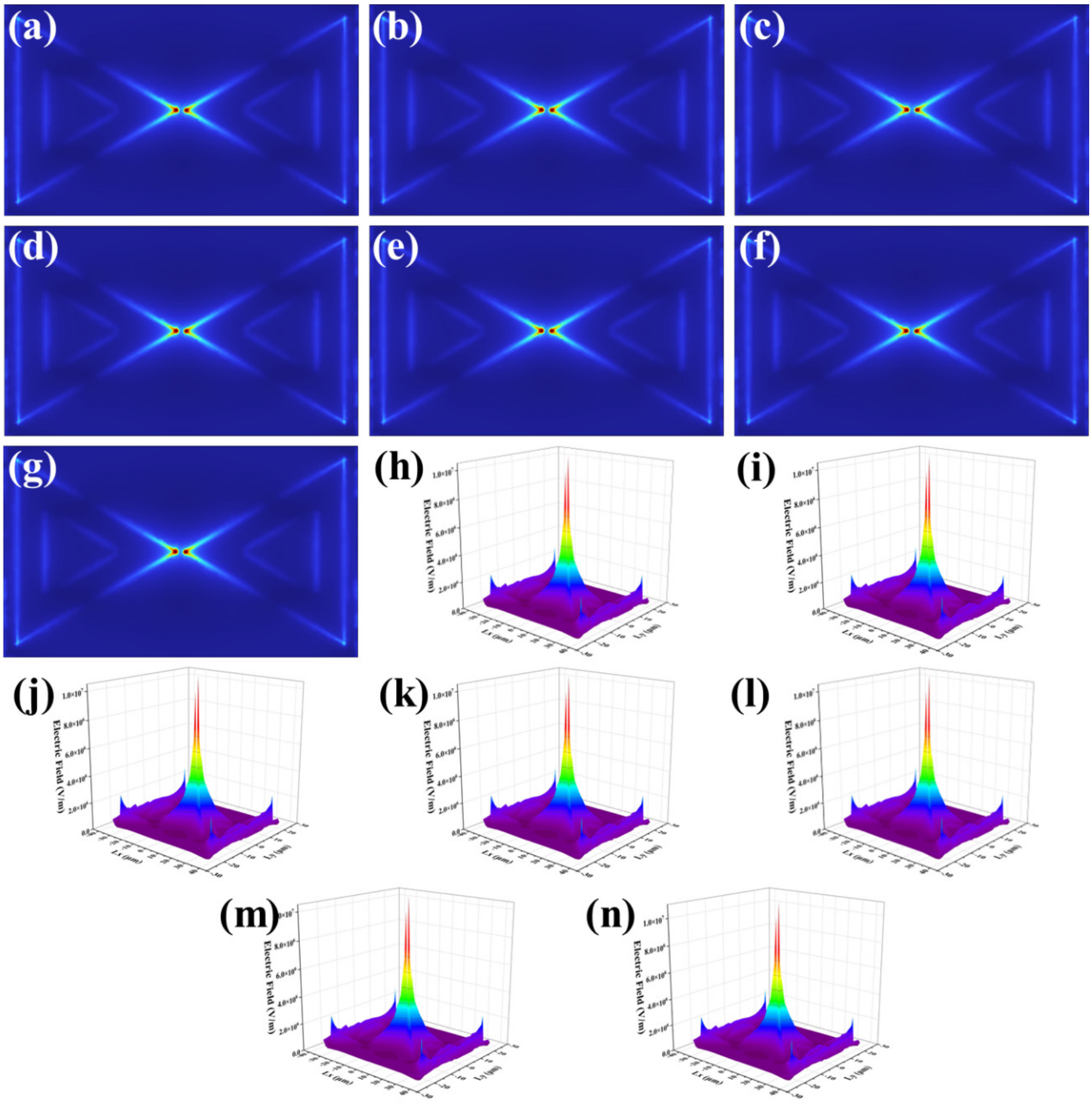

Figure S3. Distribution of electric field for the transmission valleys at different permittivity reduction. The rainbow is cut from 0 to 8×10^{6}. Notably, (a) and (h) normal blood, (b) and (i) 1% reduction, (c) and (j) 3% reduction, (d) and (k) 5% reduction, (e) and (l) 7% reduction, (f) and (m) 9% reduction, (g) and (n) 11% reduction.
